# From where to what: a neuroanatomically based evolutionary model of the emergence of speech in humans

**DOI:** 10.12688/f1000research.6175.3

**Published:** 2017-09-20

**Authors:** Oren Poliva

**Affiliations:** 1 Bangor University, Bangor, UK

**Keywords:** Speech, Evolution, Auditory dorsal stream, Contact calls, Auditory cortex, Vocal production

## Abstract

In the brain of primates, the auditory cortex connects with the frontal lobe via the temporal pole (auditory ventral stream; AVS) and via the inferior parietal lobe (auditory dorsal stream; ADS). The AVS is responsible for sound recognition, and the ADS for sound-localization, voice detection and integration of calls with faces. I propose that the primary role of the ADS in non-human primates is the detection and response to contact calls. These calls are exchanged between tribe members (e.g., mother-offspring) and are used for monitoring location. Detection of contact calls occurs by the ADS identifying a voice, localizing it, and verifying that the corresponding face is out of sight. Once a contact call is detected, the primate produces a contact call in return via descending connections from the frontal lobe to a network of limbic and brainstem regions.

Because the ADS of present day humans also performs speech production, I further propose an evolutionary course for the transition from contact call exchange to an early form of speech. In accordance with this model, structural changes to the ADS endowed early members of the genus *Homo* with partial vocal control. This development was beneficial as it enabled offspring to modify their contact calls with intonations for signaling high or low levels of distress to their mother. Eventually, individuals were capable of participating in yes-no question-answer conversations. In these conversations the offspring emitted a low-level distress call for inquiring about the safety of objects (e.g., food), and his/her mother responded with a high- or low-level distress call to signal approval or disapproval of the interaction. Gradually, the ADS and its connections with brainstem motor regions became more robust and vocal control became more volitional. Speech emerged once vocal control was sufficient for inventing novel calls.

## 1. Introduction

In the past five decades, gorillas, orangutans, chimpanzees and bonobos were shown capable of learning sign language ( Blake, 2004; Gibson, 2011). An important cognitive distinction between the language used by humans and the language used by other apes is with the ability to ask questions. This was first noted by ( Premack & Premack, 1984) who reported that, although their chimpanzee, Sarah, showed no difficulty answering questions or repeating questions before answering them, she never used the question signs for inquiring about her own environment. Jordania (2006), in his review of the literature, noted that other signing apes did not utilize questions and that their initiation of conversations was limited to commands (e.g., “me more eat”) and observational statements (e.g., “bird there”). This absence of a questioning mind is in direct contrast to human toddlers and children, who are renown for their incessant use of questions. My interpretation of this human-ape distinction is that during human evolution, we transitioned from the display of curiosity toward items that are present in our environment (i.e., observational statements) to curiosity toward items that are absent in our environment (i.e., WH questions). Developing curiosity about out of sight events and objects could thus explain the rapid migration of humans across the globe. Furthermore, this curiosity toward the unknown is the driving force behind scientific exploration and technological development. One could hence argue that it is the ability to ask that separates us from other animals and makes the human species unique.

Although no non-human primate has been reported to ask questions, they were reported to exchange calls for monitoring location (i.e., contact calls). For example, when a mother and her infant are physically separated, each emits in turn a call to signal the other their location. This emission of contact calls could therefore be interpreted as akin in meaning to the question “where are you?” If human communication and contact calls are related, it suggests that the preliminary urge to learn about the unknown is derived from infants and mothers seeking to reunite. In the present paper, based on findings collected from brain research, genetics and paleoarcheology, I demonstrate that human speech and contact calls use the same brain structures, and consequently argue that human speech emerged from contact call exchange. I then argue that by modifying their contact calls with intonations, infants were capable of signaling their mothers whether they were under high- or low-level of distress. Given the turn taking nature of these calls, and as both mothers and infants were capable of modifying their calls with intonations, the ability to choose the call type eventuated with the first yes-no conversation structure. In this scenario infants were capable of inquiring about the safety of objects in their environment (i.e., with a low-level distress call) and mothers were capable of responding to that question with a high-level distress call to signal danger or a low-level distress call to signal safety. As the use of intonations became more prevalent, conversations became more complex, and consequently vocal control became more volitional. Speech emerged once individuals acquired sufficient volitional vocal control to invent names for objects in their environment.

### 2. Models of language processing in the brain and their relation to language evolution

Throughout the 20 ^th^ century, our knowledge of language processing in the brain was dominated by the Wernicke-Lichtheim-Geschwind model ( Geschwind, 1965; Lichtheim, 1885; Wernicke, 1974). This model is primarily based on research conducted on brain-damaged individuals who were reported to possess a variety of language related disorders. In accordance with this model, words are perceived via a specialized word reception center (Wernicke’s area) that is located in the left temporoparietal junction. This region then projects to a word production center (Broca’s area) that is located in the left inferior frontal gyrus. Because almost all language input was thought to funnel via Wernicke’s area and all language output to funnel via Broca’s area, it became extremely difficult to identify the basic properties of each region. This lack of clear definition for the contribution of Wernicke’s and Broca’s regions to human language rendered it extremely difficult to identify their homologues in other primates. (For one attempt, see Aboitiz & García, 1997). With the advent of the MRI and its application for lesion mappings, however, it was shown that this model is based on incorrect correlations between symptoms and lesions and is therefore flawed ( Anderson *et al.*, 1999; DeWitt & Rauschecker, 2013; Dronkers *et al.*, 1999; Dronkers, 2000; Dronkers *et al.*, 2004; Mesulam *et al.*, 2015; Poeppel *et al.*, 2012; Vignolo *et al.*, 1986). The refutation of such an influential and dominant model opened the door to new models of language processing in the brain, and as will be presented below, to formulating a novel account of the evolutionary origins of human language from a neuroscientific perspective.

**Figure 1.**
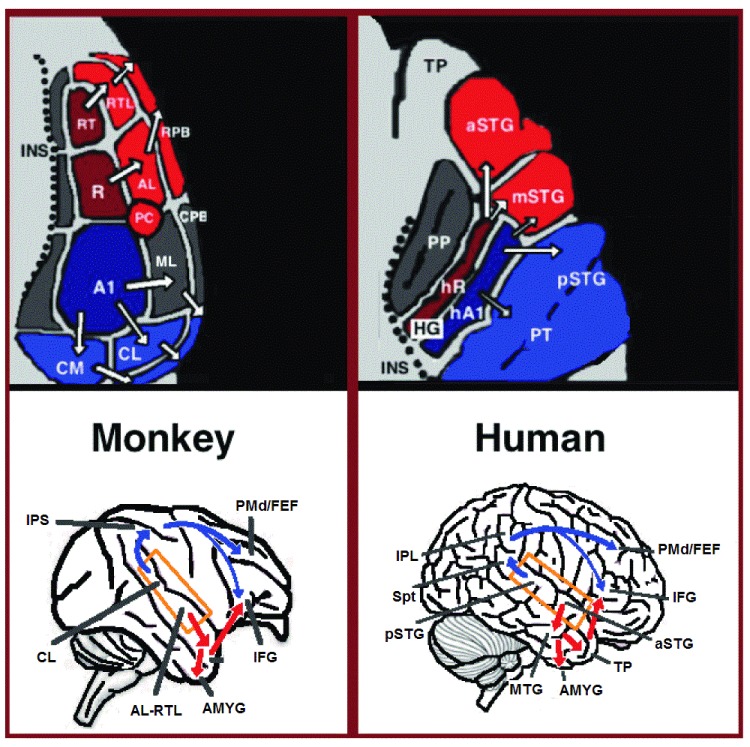
Dual stream connectivity between the auditory cortex and frontal lobe of monkeys and humans. Top: The auditory cortex of the monkey (left) and human (right) is schematically depicted on the supratemporal plane and observed from above (with the parieto-frontal operculi removed). Bottom: The brain of the monkey (left) and human (right) is schematically depicted and displayed from the side. Orange frames mark the region of the auditory cortex, which is displayed in the top sub-figures. Top and Bottom: Blue colors mark regions affiliated with the ADS, and red colors mark regions affiliated with the AVS (dark red and blue regions mark the primary auditory fields). Abbreviations: AMYG-amygdala, HG-Heschl’s gyrus, FEF-frontal eye field, IFG-inferior frontal gyrus, INS-insula, IPS-intra parietal sulcus, MTG-middle temporal gyrus, PC-pitch center, PMd-dorsal premotor cortex, PP-planum polare, PT-planum temporale, TP-temporal pole, Spt-sylvian parietal-temporal, pSTG/mSTG/aSTG-posterior/middle/anterior superior temporal gyrus, CL/ML/AL/RTL-caudo-/middle-/antero-/rostrotemporal-lateral belt area, CPB/RPB-caudal/rostral parabelt fields.

In the last two decades, significant advances occurred in our understanding of the neural processing of sounds in primates. Initially by recording of neural activity in the auditory cortices of monkeys ( Bendor & Wang, 2006; Rauschecker *et al.*, 1995) and later elaborated via histological staining ( de la Mothe *et al.*, 2006; de la Mothe *et al.*, 2012; Kaas & Hackett, 2000 - review) and fMRI scanning studies ( Petkov *et al.*, 2006), 3 auditory fields were identified in the primary auditory cortex, and 9 associative auditory fields were shown to surround them ( Figure 1 top left). Anatomical tracing and lesion studies further indicated of a separation between the anterior and posterior auditory fields, with the anterior primary auditory fields (areas R-RT) projecting to the anterior associative auditory fields (areas AL-RTL), and the posterior primary auditory field (area A1) projecting to the posterior associative auditory fields (areas CL-CM; de la Mothe *et al.*, 2006; Morel *et al.*, 1993; Rauschecker & Tian, 2000; Rauschecker *et al.*, 1997). Recently, evidence accumulated that indicates homology between the human and monkey auditory fields. In humans, histological staining studies revealed two separate auditory fields in the primary auditory region of Heschl’s gyrus ( Sweet *et al.*, 2005; Wallace *et al.*, 2002), and by mapping the tonotopic organization of the human primary auditory fields with high resolution fMRI and comparing it to the tonotopic organization of the monkey primary auditory fields, homology was established between the human anterior primary auditory field and monkey area R (denoted in humans as area hR) and the human posterior primary auditory field and the monkey area A1 (denoted in humans as area hA1; Da Costa *et al.*, 2011; Humphries *et al.*, 2010; Langers & van Dijk, 2012; Striem-Amit *et al.*, 2011; Woods *et al.*, 2010). Intra-cortical recordings from the human auditory cortex further demonstrated similar patterns of connectivity to the auditory cortex of the monkey. Recording from the surface of the auditory cortex (supra-temporal plane) reported that the anterior Heschl’s gyrus (area hR) projects primarily to the middle-anterior superior temporal gyrus (mSTG-aSTG) and the posterior Heschl’s gyrus (area hA1) projects primarily to the posterior superior temporal gyrus (pSTG) and the planum temporale (area PT; Figure 1 top right; Gourévitch *et al.*, 2008; Guéguin *et al.*, 2007). Consistent with connections from area hR to the aSTG and hA1 to the pSTG is an fMRI study of a patient with impaired sound recognition (auditory agnosia), who was shown with reduced bilateral activation in areas hR and aSTG but with spared activation in the mSTG-pSTG ( Poliva *et al.*, 2015). This connectivity pattern is also corroborated by a study that recorded activation from the lateral surface of the auditory cortex and reported of simultaneous non-overlapping activation clusters in the pSTG and mSTG-aSTG while listening to sounds ( Chang *et al.*, 2011).

Downstream to the auditory cortex, anatomical tracing studies in monkeys delineated projections from the anterior associative auditory fields (areas AL-RTL) to ventral prefrontal and premotor cortices in the inferior frontal gyrus (IFG; Muñoz *et al.*, 2009; Romanski *et al.*, 1999) and amygdala ( Kosmal *et al.*, 1997). Cortical recording and functional imaging studies in macaque monkeys further elaborated on this processing stream by showing that acoustic information flows from the anterior auditory cortex to the temporal pole (TP) and then to the IFG ( Perrodin *et al.*, 2011; Petkov *et al.*, 2008; Poremba *et al.*, 2004; Romanski *et al.*, 2005; Russ *et al.*, 2008; Tsunada *et al.*, 2011). This pathway is commonly referred to as the auditory ventral stream (AVS; Figure 1, bottom left-red arrows). In contrast to the anterior auditory fields, tracing studies reported that the posterior auditory fields (areas CL-CM) project primarily to dorsolateral prefrontal and premotor cortices (although some projections do terminate in the IFG; Cusick *et al.*, 1995; Romanski *et al.*, 1999). Cortical recordings and anatomical tracing studies in monkeys further provided evidence that this processing stream flows from the posterior auditory fields to the frontal lobe via a relay station in the intra-parietal sulcus (IPS; Cohen *et al.*, 2004; Deacon, 1992; Lewis & Van Essen, 2000; Roberts *et al.*, 2007; Schmahmann *et al.*, 2007; Seltzer & Pandya, 1984). This pathway is commonly referred to as the auditory dorsal stream (ADS; Figure 1, bottom left-blue arrows). Comparing the white matter pathways involved in communication in humans and monkeys with diffusion tensor imaging techniques indicates of similar connections of the AVS and ADS in the two species (Monkey: Schmahmann *et al.*, 2007; Human: Catani *et al.*, 2004; Frey *et al.*, 2008; Makris *et al.*, 2009; Menjot de Champfleur *et al.*, 2013; Saur *et al.*, 2008; Turken & Dronkers, 2011). In humans, the pSTG was shown to project to the parietal lobe (sylvian parietal-temporal junction-inferior parietal lobule; Spt-IPL), and from there to dorsolateral prefrontal and premotor cortices ( Figure 1, bottom right-blue arrows), and the aSTG was shown to project to the anterior temporal lobe (middle temporal gyrus-temporal pole; MTG-TP) and from there to the IFG ( Figure 1 bottom right-red arrows).

On the basis of converging evidence collected from monkeys and humans, it has been established that the AVS is responsible for the extraction of meaning from sounds (see appendix A for a review of the literature). Specifically, the anterior auditory cortex is ascribed with the perception of auditory objects, and downstream, the MTG and TP are thought to match the auditory objects with their corresponding audio-visual semantic representations (i.e., the semantic lexicon). This recognition of sounds in the AVS, although critical for intact communication, appears to contribute less to the uniqueness of human language than the ADS. This is demonstrated by the universality of sound recognition, as many mammalian species use it for identifying prey, predators or potential mates. As an example, dogs were reported capable of recognizing spoken words and extract their meaning ( Kaminski *et al.*, 2004; Pilley & Reid, 2011), and with fMRI this sound recognition ability was localized to the TP of the AVS ( Andics *et al.*, 2014). Studies also provided evidence that the sound recognition of non-human apes is equivalent in complexity to ours. Apes trained in human facilities were reported capable of learning human speech and comprehending its meaning (e.g., the bonobos, Kanzi and Panbanisha, were reported to recognize more than 3000 spoken English words; Blake, 2004; Gibson, 2011). Moreover, a study that compared humans and a chimpanzee in their recognition of acoustically distorted spoken words, reported no differences between chimpanzee and human performance ( Heimbauer *et al.*, 2011).

In contrast to the relatively preserved function of the AVS among mammals, converging evidence suggests that the ADS was significantly modified since our *Hominin* ancestors separated from other apes. For instance, a diffusion tensor imaging study that compared the white matter of humans and chimpanzees demonstrated significant strengthening of ADS connectivity, but not AVS connectivity ( Rilling *et al.*, 2012). Evidence for restructuring of the ADS during *Hominin* evolution is also demonstrated in the fossil record. A study that reconstructed the endocranium of early *Hominins* noted that *Homo habilis*, but not any of its *Australopith* ancestors, is characterized by a dramatic heightening of the IPL and enlargement (though to a lesser degree) of the IFG, whereas the rest of the endocranium remains extremely similar to the endocranium of modern apes ( Tobias, 1987). It is also worth reporting that the recently discovered *Australopithecus sediba* ( Carlson *et al.*, 2011), which is the closest known relative to the *Australopith* predecessor of *Homo habilis*, is characterized with a very ape-like parietal and frontal lobes (although some modifications of the orbitofrontal surface were noted). These findings also suggest that it was changes to the ADS that initially prompted the brain enlargement that characterized *Hominans* (members of the genus *Homo;* Wood & Richmond, 2000), and separated us from other *Hominins.*

In contrast to the AVS, the ADS was ascribed with a diverse range of seemingly unrelated functions. These functions, which will be detailed throughout this paper, include auditory localization, audio-visual integration, and voice detection in monkeys. In humans, the ADS has been further ascribed with the preparation and production of speech. In the present paper, based on functional differences between the ADS of monkeys and humans, I propose intermediate stages in the development of human speech.

### 3. The role of the ADS in audiospatial processing

The most established role of the ADS is with audiospatial processing. This is evidenced via studies that recorded neural activity from the auditory cortex of monkeys, and correlated the strongest selectivity to changes in sound location with the posterior auditory fields (areas CM-CL), intermediate selectivity with primary area A1, and very weak selectivity with the anterior auditory fields ( Benson *et al.*, 1981; Miller & Recanzone, 2009; Rauschecker *et al.*, 1995; Tian *et al.*, 2001; Woods *et al.*, 2006). In humans, behavioral studies of brain damaged patients ( Clarke *et al.*, 2000; Griffiths *et al.*, 1996) and EEG recordings from healthy participants ( Anourova *et al.*, 2001) demonstrated that sound localization is processed independently of sound recognition, and thus is likely independent of processing in the AVS. Consistently, a working memory study ( Clarke *et al.*, 1998) reported two independent working memory storage spaces, one for acoustic properties and one for locations. Functional imaging studies that contrasted sound discrimination and sound localization reported a correlation between sound discrimination and activation in the mSTG-aSTG, and correlation between sound localization and activation in the pSTG and PT ( Ahveninen *et al.*, 2006; Alain *et al.*, 2001; Barrett & Hall, 2006; De Santis *et al.*, 2007; Viceic *et al.*, 2006; Warren & Griffiths, 2003), with some studies further reporting of activation in the Spt-IPL region and frontal lobe ( Hart *et al.*, 2004; Maeder *et al.*, 2001; Warren *et al.*, 2002). Some fMRI studies also reported that the activation in the pSTG and Spt-IPL regions increased when individuals perceived sounds in motion ( Baumgart *et al.*, 1999; Krumbholz *et al.*, 2005; Pavani *et al.*, 2002). EEG studies using source-localization also identified the pSTG-Spt region of the ADS as the sound localization processing center ( Tata & Ward, 2005a; Tata & Ward, 2005b). A combined fMRI and MEG study corroborated the role of the ADS with audiospatial processing by demonstrating that changes in sound location resulted in activation spreading from Heschl’s gyrus posteriorly along the pSTG and terminating in the IPL ( Brunetti *et al.*, 2005). In another MEG study, the IPL and frontal lobe were shown active during maintenance of sound locations in working memory ( Lutzenberger *et al.*, 2002).

In addition to localizing sounds, the ADS appears also to encode the sound location in memory, and to use this information for guiding eye movements. Evidence for the role of the ADS in encoding sounds into working memory is provided via studies that trained monkeys in a delayed matching to sample task, and reported of activation in areas CM-CL ( Gottlieb *et al.*, 1989) and IPS ( Linden *et al.*, 1999; Mazzoni *et al.*, 1996) during the delay phase. Influence of this spatial information on eye movements occurs via projections of the ADS into the frontal eye field (FEF; a premotor area that is responsible for guiding eye movements) located in the frontal lobe. This is demonstrated with anatomical tracing studies that reported of connections between areas CM-CL-IPS and the FEF ( Cusick *et al.*, 1995; Stricanne *et al.*, 1996), and electro-physiological recordings that reported neural activity in both the IPS ( Linden *et al.*, 1999; Mazzoni *et al.*, 1996; Mullette-Gillman *et al.*, 2005; Stricanne *et al.*, 1996) and the FEF ( Russo & Bruce, 1994; Vaadia *et al.*, 1986) prior to conducting saccadic eye-movements toward auditory targets.

### 4. The role of the ADS in the localization of con-specifics

In addition to processing the locations of sounds, evidence suggests that the ADS further integrates sound locations with auditory objects. Demonstrating this integration are electrophysiological recordings from the posterior auditory cortex ( Recanzone, 2008; Tian *et al.*, 2001) and IPS ( Gifford & Cohen, 2005), as well a PET study ( Gil-da-Costa *et al.*, 2006), that reported neurons that are selective to monkey vocalizations. One of these studies ( Tian *et al.*, 2001) further reported neurons in this region (CM-CL) that are characterized with dual selectivity for both a vocalization and a sound location. Consistent with the role of the pSTG-PT in the localization of specific auditory objects are also studies that demonstrate a role for this region in the isolation of specific sounds. For example, two functional imaging studies correlated circumscribed pSTG-PT activation with the spreading of sounds into an increasing number of locations ( Smith *et al.*, 2010-fMRI; Zatorre *et al.*, 2002-PET). Accordingly, an fMRI study correlated the perception of acoustic cues that are necessary for separating musical sounds (pitch chroma) with pSTG-PT activation ( Warren *et al.*, 2003).

When elucidating the role of the primate ADS in the integration of a sound’s location with calls, it remains to be determined what kind of information the ADS extracts from the calls. This information could be then used to make inferences about the function of the ADS. Studies from both monkeys and humans suggest that the posterior auditory cortex has a role in the detection of a new speaker. A monkey study that recorded electrophysiological activity from neurons in the posterior insula (near the pSTG) reported neurons that discriminate monkey calls based on the identity of the speaker ( Remedios *et al.*, 2009a). Accordingly, human fMRI studies that instructed participants to discriminate voices reported an activation cluster in the pSTG ( Andics *et al.*, 2010; Formisano *et al.*, 2008; Warren *et al.*, 2006). A study that recorded activity from the auditory cortex of an epileptic patient further reported that the pSTG, but not aSTG, was selective for the presence of a new speaker ( Lachaux *et al.*, 2007-patient 1). The role of this posterior voice area, and the manner in which it differs from voice recognition in the AVS ( Andics *et al.*, 2010; Belin & Zatorre, 2003; Nakamura *et al.*, 2001; Perrodin *et al.*, 2011; Petkov *et al.*, 2008), was further shown via electro-stimulation of another epileptic patient ( Lachaux *et al.*, 2007-patient 2). This study reported that electro-stimulation of the aSTG resulted in changes in the perceived pitch of voices (including the patient’s own voice), whereas electro-stimulation of the pSTG resulted in reports that her voice was “drifting away.” This report indicates a role for the pSTG in the integration of sound location with an individual voice. Consistent with this role of the ADS is a study that reported patients, with AVS damage but spared ADS (surgical removal of the anterior STG/MTG), were no longer capable of isolating environmental sounds in the contralesional space, whereas their ability of isolating and discriminating human voices remained intact ( Efron & Crandall, 1983). Preliminary evidence from the field of fetal cognition suggests that the ADS is capable of identifying voices in addition to discriminating them. By scanning fetuses of third trimester pregnant mothers with fMRI, the researchers reported of activation in area Spt when the hearing of voices was contrasted to pure tones ( Jardri *et al.*, 2012). The researchers also reported that a sub-region of area Spt was more selective to maternal voice than unfamiliar female voices. Based on these findings, I suggest that the ADS has acquired a special role in primates for the localization of conspecifics.

### 5. The role of the ADS in the detection of contact calls

To summarize, I have argued that the monkey’s ADS is equipped with the algorithms required for detecting a voice, isolating the voice from the background cacophony, determining its location, and guiding eye movements for the origin of the call. An example of a behavior that utilizes all these functions is the exchange of contact calls, which are used by extant primates to monitor the location or proximity of conspecific tribe members ( Biben *et al.*, 1986; Sugiura, 1998). The utilization of these ADS functions during the exchange of contact calls was demonstrated in studies of squirrel monkeys and vervet monkeys ( Biben, 1992; Biben *et al.*, 1989; Cheney & Seyfarth, 1980; Symmes & Biben, 1985). In both species, mothers showed no difficulty in isolating their own infant’s call, localizing it, and maintaining this location in their memory while approaching the source of the sound. A similar use of contact calls has been documented in our closest relatives, chimpanzees. The exchange of pant-hoot calls was documented between chimpanzees that were separated by great distances ( Goodall, 1986; Marler & Hobbett, 1975) and was used for re-grouping ( Mitani & Nishida, 1993). Because infants respond to their mother’s pant-hoot call with their own unique vocalization (staccato call; Matsuzawa, 2006), the contact call exchange appears also to play an important role in the ability of mothers to monitor the location of their infants. It is also worth noting that when a chimpanzee produced a pant-hoot call and heard no call in response, the chimpanzee was reported to carefully scan the forest before emitting a second call ( Goodall, 1986). This behavior demonstrates the relationship between the detection of contact calls, the embedding of auditory locations in a map of the environment, and the guidance of the eyes for searching the origin of the call. Further corroborating the involvement of the ADS in the detection of contact calls are intra-cortical recordings from the posterior insula (near area CM-A1) of the macaque, which revealed stronger selectivity for a contact call (coo call) than a social call (threat call; Remedios *et al.*, 2009a). Contrasting this finding is a study that recorded neural activity from the anterior auditory cortex, and reported that the proportion of neurons dedicated to a contact call was similar to the proportions of neurons dedicated to other calls ( Perrodin *et al.*, 2011).

**Figure 2.**
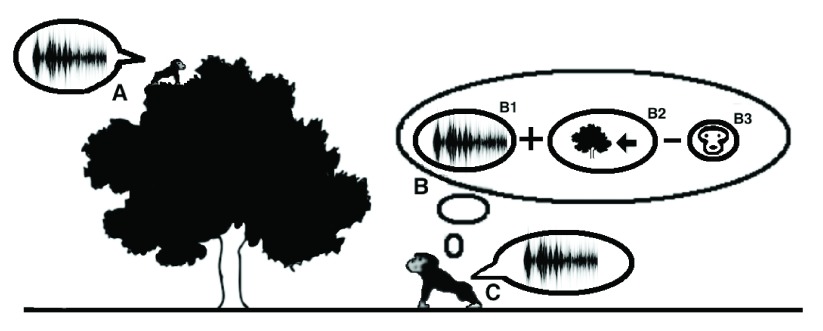
Discrete stages in contact call exchange. In accordance with the model, the original function of the ADS is for the localization of and the response to contact calls that are exchanged between mothers and their infants. When an infant emits a contact call ( **A**), the mother identifies her offspring’s voice (B1) localizes the call (B2) and maintains this information in visual working memory. Then, if the corresponding face is absent in that location (B3), the mother emits a contact call in return ( **C**).

Perceiving a contact call can be viewed as a three-step process. The individual is required to detect a voice, integrate it with its location and verify that no face is visible in that location ( Figure 2). In the previous paragraphs, I provided evidence for the involvement of the ADS in the first two stages (voice detection and localization). Evidence for the role of the ADS in the integration of faces with their appropriate calls is provided by a study that recorded activity from the monkey auditory cortex (areas A1 and ML; Ghazanfar *et al.*, 2005). The monkeys were presented with pictures of a monkey producing a call in parallel to hearing the appropriate call, or only saw the face or heard the call in isolation. Consistent with the prediction from the present model that visual inspection of faces inhibits processing of contact calls, the face-call integration was much more enhanced for the social call (grunt call) than for the contact call (coo call). Associating this integration of faces with calls with processing in the ADS is consistent with a monkey fMRI study that correlated audio-visual integration with activation in the posterior, but not in the anterior, auditory fields ( Kayser *et al.*, 2009).

### 6. The role of the ADS in the response to contact calls

Hitherto, I have argued that the ADS is responsible for the perception of contact calls. However, as the perception of a contact call leads to producing a contact call in return, it is also desirable to suggest a pathway through which the ADS mediates vocal production.

Cumulative evidence suggests that most vocalizations in non-human primates are prepared and produced in a network of limbic and brainstem regions, and do not appear to be controlled by the ADS. For instance, studies that damaged the temporoparietal and/or the IFG regions of monkeys reported that such lesions had no effect on spontaneous vocal production ( Aitken, 1981; Sutton *et al.*, 1974). This conclusion is also consistent with comprehensive electro-stimulation mappings of the monkey’s brain, which reported no spontaneous vocal production during stimulation of the temporal, occipital, parietal, or frontal lobes ( Jürgens & Ploog, 1970; Robinson, 1967). These studies, however, reported emission of vocalizations after stimulating limbic and brainstem regions (amygdala, anterior cingulate cortex, basal forebrain, hypothalamus, mid-brain periaqueductal gray). Moreover, based on a study that correlated chemical activation in the mid-brain periaqueductal gray with vocal production, it was inferred that all the limbic regions project to central pattern generators in the periaqueductal gray, which orchestrates the vocal production ( Zhang *et al.*, 1994). In a series of tracing studies and electrophysiological recordings, it was also shown that the periaqueductal gray projects to pre-motor brainstem areas ( Hage & Jürgens, 2006; Hannig & Jürgens, 2006), which in turn project to brainstem motor nuclei ( Holstege, 1989; Holstege *et al.*, 1997; Lüthe *et al.*, 2000; Vanderhorst *et al.*, 2000; Vanderhorst *et al.*, 2001). The brainstem motor nuclei then directly stimulate the individual muscles of the vocal apparatus. Because documented calls of non-human primates (including chimpanzees) were shown with very little plasticity ( Arcadi, 2000) and were observed only in highly emotional situations ( Goodall, 1986), these limbic-brainstem generated calls are likely more akin to human laughter, sobbing, and screaming than to human speech.

Although most monkey vocalizations can be produced without cortical control, some calls, such as alarm calls and contact calls are context dependent and are thus likely under cortical influence ( Biben, 1992; Seyfarth *et al.*, 1980). Furthermore, several studies demonstrated that contact calls are directly regulated by the ADS. For instance, a study that recorded neural activity from the IFG of macaques reported of neural discharge prior to cued or spontaneous contact call production (coo calls), but not prior to production of vocalizations-like facial movements (i.e., silent vocalizations; Coudé *et al.*, 2011; see also Gemba *et al.*, 1999 for similar results). Consistently, a study that sacrificed marmoset monkeys immediately after responding to contact calls (phee calls) measured highest neural activity (genomic expression of cFos protein) in the posterior auditory fields (CM-CL), and IFG ( Miller *et al.*, 2010). Monkeys sacrificed after only hearing contact calls or only emitting them showed neural activity in the same regions but to a much smaller degree (See also Simões *et al.*, 2010 for similar results in a study using the protein Egr-1). Anatomical tracing studies ( Jürgens & Alipour, 2002; Roberts *et al.*, 2007) demonstrated direct connections from the IFG of monkeys to limbic and brainstem regions, thus providing a possible route for controlling the contact call response. The former study ( Jürgens & Alipour, 2002), however, further reported of a second direct connection from the IFG to a brainstem motor nucleus (hypglossal nucleus) which controls tongue movements. Although the role of this pathway is not yet known, its anatomical connectivity implies that it is capable of bypassing the limbic-brainstem vocal network, and provides some volitional control over the vocal apparatus. This conclusion is further consistent with behavioral studies of monkeys that reported partial volitional control in the contact call response. For instance, a study that followed macaque mothers and babies reported that the macaque mothers were capable, to a limited extent, of modifying their contact calls to acoustically match those of their infants ( Masataka, 2009). Squirrel monkeys and macaque monkeys were also reported to modify the frequencies of their contact calls, which resulted with the caller and responder emitting slightly different calls ( Biben *et al.*, 1986; Sugiura, 1998). In one study, macaque monkeys were even observed to spontaneously modify the vocal properties of their contact call for requesting different objects from the experimenter ( Hihara *et al.*, 2003). Anecdotal reports of more generalized volitional vocal control, albeit rudimentary, in apes ( Hayes & Hayes, 1952; Hopkins *et al.*, 2007; Kalan *et al.*, 2015; Koda *et al.*, 2007; Koda *et al.*, 2012; Lameira *et al.*, 2015; Laporte & Zuberbühler, 2010; Perlman & Clark, 2015; Taglialatela *et al.*, 2003; Wich *et al.*, 2008) suggest that the direct connections between the IFG and the brainstem motor nuclei were strengthened prior to our divergence from our apian relatives.

### 7. From contact calls to speech

In the previous sections I provided evidence that the ADS of non-human primates is responsible for the detection and response to contact calls. In the present section I present converging evidence that in humans the ADS performs speech production, and argue that human speech emerged from the exchange of contact calls.

Evidence for a role of the ADS in the transition from mediating contact calls into mediating human speech includes genetic studies that focused on mutation to the protein SRPX2 and its regulator protein FOXP2 ( Roll *et al.*, 2010). In mice, blockage of SRPX2 or FOXP2 genes resulted in pups not emitting distress calls when separated from their mothers ( Shu *et al.*, 2005; Sia *et al.*, 2013). In humans, however, individuals afflicted with a mutated SRPX2 or FOXP2 were reported with speech dyspraxia ( Roll *et al.*, 2006; Watkins *et al.*, 2002). A PET imaging study of an individual with a mutated SRPX2 gene correlated this patient’s disorder with abnormal activation (hyper-metabolism) along the ADS (pSTG-Spt-IPL; Roll *et al.*, 2006). Similarly, an MRI study that scanned individuals with mutated FOXP2 reported increased grey matter density in the pSTG-Spt and reduced density in the IFG, thus further demonstrating abnormality in ADS‘ structures ( Belton *et al.*, 2003). A role for the ADS in mediating speech production in humans has also been demonstrated in studies that correlated a more severe variant of this disorder, apraxia of speech, with IPL and IFG lesions ( Deutsch, 1984; Edmonds & Marquardt, 2004; Hillis *et al.*, 2004; Josephs *et al.*, 2006; Kimura & Watson, 1989; Square *et al.*, 1997). The role of the ADS in speech production is also demonstrated via a series of studies that directly stimulated sub-cortical fibers during surgical operations ( Duffau, 2008-review), and reported that interference in the left pSTG and IPL resulted in an increase in speech production errors, and interference in the left IFG resulted in speech arrest (see also Acheson *et al.*, 2011; Stewart *et al.*, 2001 for similar results using magnetic interference in healthy individuals). One study even reported that stimulation of the left IPL resulted with patients believing that they spoke, when they didn’t, and IFG stimulation resulted with the patients unconsciously moving their lips ( Desmurget *et al.*, 2009).

Further support for the transition from contact call exchange to human speech are provided by studies of hemispheric lateralization ( Petersen *et al.*, 1978). In one study, Japanese macaques and other old world monkeys were trained to discriminate contact calls of Japanese macaques, which were presented to the right or left ear. Although all the monkeys were capable of completing the task, only the Japanese macaques were noted with right ear advantage, thus indicating left hemispheric processing of contact calls. In a study replicating the same paradigm, Japanese macaques had an impaired ability to discriminate contact calls after suffering unilateral damage to the auditory cortex of the left, but not right, hemisphere ( Heffner & Heffner, 1984). This leftward lateralization of contact call detection is similar to the long established role of the human left hemisphere in the processing human language ( Geschwind, 1965).

### 8. Prosodic speech and the emergence of conversations

A possible route for the transition from contact call exchange to proto-speech was proposed by Dean Falk (2004). She argued that due to bipedal locomotion and the loss of hair in early *Hominins*, mothers were not capable of carrying their infants while foraging. As a result, the mothers maintained contact with their infant through a vocal exchange of calls that resembles contemporary “motherese” (the unique set of intonations that caregivers use when addressing infants). As previously suggested by another researcher ( Masataka, 2009), such intermediate prosodic phase in the development of speech is consistent with evidence (presented in section 5) that monkeys, to a limited extent, are capable of modifying their contact calls with intonations, and that apes are endowed with slightly more versatile vocal control. In the context of the present model, such evolutionary course implies that throughout *Hominan* evolution, the ADS gained increased control over the vocal apparatus, possibly by strengthening the direct connections of the IFG with the brainstem motor nuclei. Consistent with this view, many studies demonstrated a role for the ADS in the perception and production of intonations. For instance, an fMRI study that instructed participants to rehearse speech, reported that perception of prosodic speech, when contrasted with flattened speech, results in a stronger activation of the PT-pSTG of both hemispheres ( Meyer *et al.*, 2004). In congruence, an fMRI study that compared the perception of hummed speech to natural speech didn’t identify any brain area that is specific to humming, and thus concluded that humming is processed in the speech network ( Ischebeck *et al.*, 2004). fMRI studies that instructed participants to analyze the rhythm of speech also reported of ADS activation (Spt, IPL, IFG; Geiser *et al.*, 2008; Gelfand & Bookheimer, 2003). An fMRI study that compared speech perception and production to the perception and production of humming noises, reported in both conditions that the overlapping activation area for perception and production (i.e., the area responsible for sensory-motor conversion) was located in area Spt of the ADS ( Hickok *et al.*, 2003). Supporting evidence for the role of the ADS in the production of prosody are also studies reporting that patients diagnosed with apraxia of speech are additionally diagnosed with expressive dysprosody ( Odell *et al.*, 1991; Odell & Shriberg, 2001; Shriberg *et al.*, 2006 - FOXP2 affected individuals). Finally, the evolutionary account proposed here from vocal exchange of calls to a prosodic-based language is similar to the recent development of whistling languages, since these languages were documented to evolve from exchanging simple calls used to report speakers’ locations into a complex semantic system based on intonations ( Meyer, 2008).

**Figure 3.**
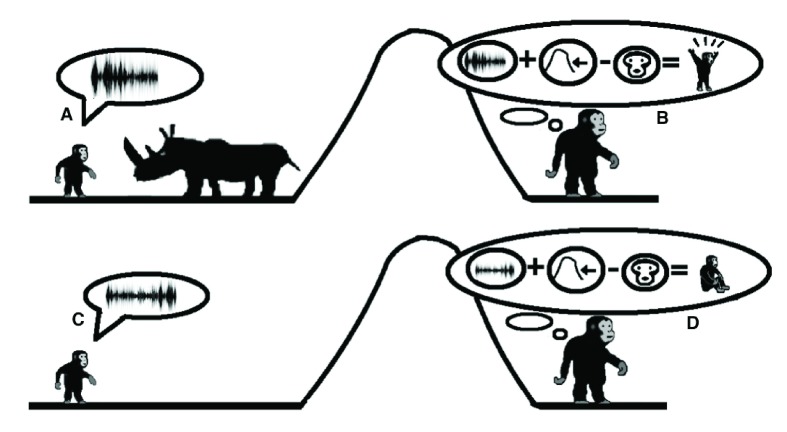
The use of prosody to signal levels of distress. In accordance with the model, *early* Hominans became capable of modifying their contact calls with intonations (prosody). This modification could have originated for the purpose of expressing different levels of distress. In this figure, we see a *Homo habilis* child using prosody to modify the contact call to express a high level of distress ( **A**) or a low level of distress ( **C**). The child’s mother then registers the call (by integrating his prosodic intonation and voice, location, and the absence of his face) to recognize whether her child requires immediate ( **B**) or non-immediate ( **D**) attention.

In the opening paragraph of this paper, I described the inability of apes to ask questions, and proposed that the ability to ask questions emerged from contact calls. Because the ability to ask questions likely co-emerged with the ability to modify calls with prosodic intonations, I expand Falk’s and Masataka’s views regarding the prosodic origins of vocal language, and propose that the transition from contact calls to prosodic intonations could have emerged as a means of enabling infants to express different levels of distress ( Figure 3). In such a scenario, the modification of a call with intonations designed to express a high level of distress is akin in meaning to the sentence “mommy, come here now!”. Hence, the modification of calls with intonations could have served as a precursor for the development of prosody in contemporary vocal commands. On the other hand, the use of intonations for expressing a low-level of distress is akin in meaning to the sentence “mommy, where are you?”. Therefore, this use of prosody for asking the first question could have served as the precursor for pragmatically converting calls into questions by using prosody as well. This transition could be related to the ability of present-day infants of using intonations for changing the pragmatic utilization of a word from a statement to a command/demand (“MOMMY!”) or a question (“mommy?”). This view is consistent with a longitudinal developmental study of toddlers, which reported of the toddlers utilizing prosodic intonations in their speech prior to construction of sentences ( Snow, 1994). A study of speech perception in adults also demonstrated that our ability to discriminate questions sentences from statement sentences is dependent on analysis of prosodic intonations ( Srinivasan *et al.*, 2003). Evidence of the relationship between the ability to ask questions and processing in the ADS is demonstrated in a diffused tensor imaging and fMRI study ( Sammler *et al.*, 2015), which reported the participation of both the ADS and AVS in the discrimination of mono-syllabic words into questions or statements. The researchers further showed that this discrimination was impaired while interference was induced with TMS in the pre-motor cortex of the ADS. Supporting the role of the ADS in the discrimination of questions and statements is the finding that patients with phonological dementia, who are known to suffer from degeneration along the ADS ( Gorno-Tempini *et al.*, 2008; Rohrer *et al.*, 2010), were impaired in distinguishing whether a spoken word was a question or a statement ( Rohrer *et al.*, 2012).

**Figure 4.**
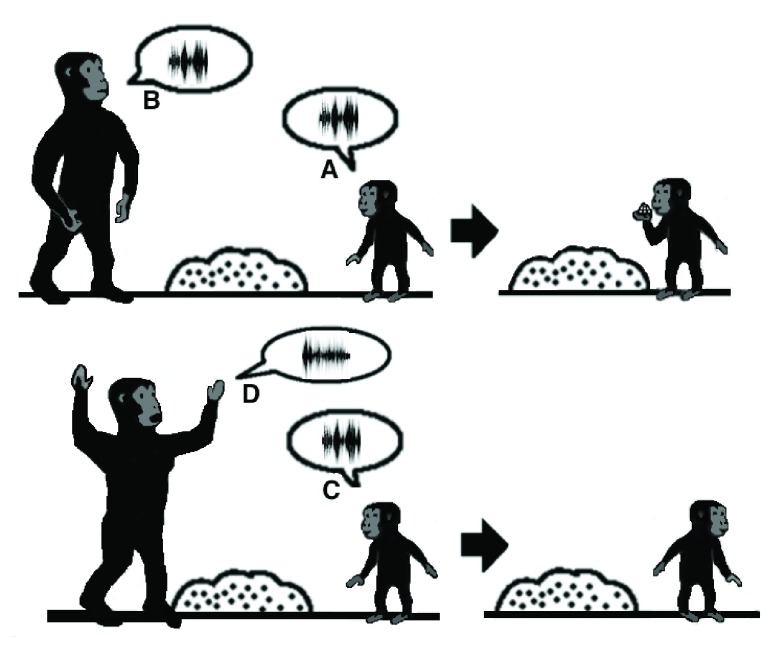
Prosody and the emergence of question-answer conversations. In accordance with the model, the modification of contact calls with intonations for reporting distress levels eventually transitioned into question-answer conversations about items in their environment. In this figure, a child is using low-level distress call ( **A**, **C**) to ask permission to eat an unfamiliar food (berries). The mother can then respond with a high-level distress call ( **D**) that signals danger or a low-level distress ( **B**) that signals safety.

A possible route for the transition from emitting low-level distress calls to asking questions is by individuals starting to utilize the former to signal interest about objects in their environment. Given that both contact call exchange and contemporary speech are characterized with turn taking, early *Hominans* could have responded to the low-level distress calls with either high- or low-level distress calls. For example, when an infant expressed a low-level distress call prior to eating berries, his/her mother could have responded with a high-level distress call that indicated the food is dangerous or a low-level distress call that indicated the food is safe ( Figure 4). Eventually, the infant emitted the question call and waited for an appropriate answer from their mother before proceeding with their intended action. This conversation structure could be the precursor to present-day yes/no questions.

The proto-conversations described so far are very limited in their content as the meaning of each call is dependent on context. In order for speech to become more versatile, early *Hominans* needed a method for acquiring vocabulary. A possible route for the acquisition of words is that the prevalence of using intonations gradually resulted with increase in volitional control over the vocal apparatus. Eventually, vocal control was sufficient for inventing novel calls. Offspring, which so far communicated vocally with their parents for signaling interest in interacting with objects, began mimicking their parents’ vocal response. Eventually, by practicing mimicry, the offspring learned the names of objects and enhanced their vocabulary. Transitioning to children demonstrating curiosity for the names of objects could have also prompted the curiosity towards the unknown that characterizes our species. This period of mimicry in language development could be the reason present day babies constantly mimic their parents’ vocalizations. In depth discussion about the role of vocal mimicry in language development and its relation with the ADS is beyond the scope of the present paper. However, an evolutionary account of the emergence of language from mimicry based conversations and its relation with the ADS and AVS is discussed in detail in a follow up paper, titled ‘From Mimicry to Language: A Neuroanatomically Based Evolutionary Model of the Emergence of Vocal Language’ ( Poliva, 2016).

### 9. Comparisons of the ‘From Where to What’ model to previous language evolution models

Following in the footsteps of Dean Falk and Nobuo Masataka, the present model argues that human speech emerged from the exchange of contact calls via a transitory prosodic phase. Since the principle of natural selection was first acknowledged by the scientific community however, several other accounts of language evolution were proposed. Here, I’ll present two schools of thought, and discuss their validity in the context of the present model.

The earliest model for language evolution was proposed by Charles Darwin. In his book, *The Descent of Man* (1871), Darwin equated speech exchange to bird song, and proposed that the perception and production of songs during mating rituals were the precursor to human language (singing ape hypothesis). Similar accounts suggesting music to participate in the evolutionary development of speech were also proposed by more recent researchers ( Jordania, 2006; Masataka, 2009; Mithen, 2006). However, so far the idea of music as precursor to language has not taken hold in the scientific community due to lack of substantiating evidence. In appendix A, I cite evidence that the perception of melodies occurs in the aSTG of the AVS. Given the mounting evidence indicating that speech is processed primarily in the ADS, we would expect that precursors to speech would also be processed in the same pathway (although, see the review by Stewart *et al.*, 2006 who suggests roles also for other auditory fields in music perception). Since I hypothesize that singing-like calls were utilized for communication prior to complex vocal language, the idea of music perception and production isn’t too different from the present model. However, arguing that music served as precursor to speech is different than arguing that music and speech emerged from a common proto-function. Investigating whether music served as a precursor to vocal language is problematic since such a model implies that music perception is a unique human trait. Therefore, in order to resolve the conundrum of music evolution and its level of contribution to the emergence of vocal language, future studies should first attempt to determine whether non-human primates can perceive music. (See Remedios *et al.*, 2009b for preliminary findings).

A more recent school of thought argues that language with complex semantics and grammar was first communicated via the exchange of gestures and only recently became vocal (Gestural language model; Arbib, 2008; Corballis, 2010; Donald, 2005; Gentilucci & Corballis, 2006; Hewes, 1973; Studdert-Kennedy, 2005). In accordance with this model, speech could have served for increasing communication distance and enabling communication under low visibility conditions (e.g., night, caves). This model is primarily based on the natural use of gestural communication between non-human primates, the ability of apes to learn sign language, and the natural development of sign languages in deaf communities. This model also received increased popularity since the discovery of mirror neurons, as these neurons are interpreted by proponents of the model as evidence of a mechanism dedicated to the imitation of gestures. From a neuroanatomical perspective it is plausible that vocal communication emerged from gestures. For instance, an fMRI study correlated hearing animal calls with bilateral activation in the mSTG-aSTG, whereas hearing manual tool sounds (e.g., hammer, saw) correlated with activation in the pSTG and IPL of the hemisphere contralateral to the dominant hand ( Lewis *et al.*, 2006). This recognition of tool sounds in the ADS instead of AVS is surprising because it could suggest that the teaching of tool use, which required gestures, was associated with speech production. This view is also supported by a study that reported of an area that is co-selective to the detection of hands and manual tools (i.e., area responsible for the perception of tool usage), which is located near the pSTG ( Bracci *et al.*, 2012), and not in the area most often responsible for visual object recognition, the inferior temporal lobe. Finally, it is interesting to note that damage to the ADS (areas Spt, IPL and IFG) in the left hemisphere were strongly associated with errors gesturing tool use ( Manuel *et al.*, 2013). Based on these findings I find the hypothesis that speech and gestures co-evolved compelling. However, given that my model delineates a course for the development of proto-conversations from calls that are used by extant primates, it is incongruent with the argument that a gestural language with complex grammar and semantics preceded vocal language.

### 10. ‘From Where to What’- Future Research

In the present paper, I delineate a course for the early development of language by proposing four hypotheses: 1. In non-human primates, the ADS is responsible for perceiving and responding to contact calls; 2. Mother-offspring vocal exchange was the predominant force that guided the emergence of speech in the ADS; 3. Speech emerged from modifying calls with intonations for signaling a low-level and high-level of distress, and these calls are the precursor to our use of intonations for converting words into questions and commands, respectively. 4. Asking questions is a unique human characteristic and the primary driving force behind our species’ cognitive success. Cumulative and converging evidence for the veracity of each of these hypotheses was provided throughout the paper. However, as the veracity of a model can only be measured by its ability to predict experimental results, I will present here outlines for 4 potential studies that can test each of these hypotheses.

In accordance with the first hypothesis, the ADS of non-human primates is responsible for the detection and vocal response to contact calls. A possible way of testing this hypothesis is by inducing bilateral lesions to the temporo-parietal junction of a monkey and then measuring whether the monkey no longer responds vocally to contact calls or responds less than before the lesion induction.

In accordance with the second hypothesis, mother infant interaction was the guiding force that endowed the ADS with its role in speech. This hypothesis is primarily based on the finding that a sub-region of area Spt in human fetuses was shown selective to the voice of their mothers ( Jardri *et al.*, 2012). Future studies should further explore whether this region remains active in the brain of infants and toddlers and whether mothers also possess a region in the ADS that is selective to the voice of their children.

In accordance with the third hypothesis, the ADS originally served for discriminating calls that signal different levels of distress by analyzing their intonations. At present day, this development is reflected in our ability to modify intonations for converting spoken words into questions and commands. A way of testing this hypothesis is by using fMRI to compare the brain regions active when participants discriminate spoken words into questions and commands, with the brain regions active when they discriminate these words based on their emotional content (e.g., scared and happy). I predict that the former will activate the ADS whereas the latter the AVS.

In accordance with the fourth hypothesis, the unique human mind is the result of our ability to ask questions. To test whether this hypothesis is true, when teaching apes sign language, more effort should be allocated in training them to ask questions.

## Appendix A: The auditory ventral stream and its role in sound recognition

Accumulative converging evidence indicates that the AVS is involved in recognizing auditory objects. At the level of the primary auditory cortex, recordings from monkeys showed higher percentage of neurons selective for learned melodic sequences in area R than area A1 ( Yin *et al.*, 2008), and a study in humans demonstrated more selectivity for heard syllables in the anterior Heschl’s gyrus (area hR) than posterior Heshcl’s gyrus (area hA1; Steinschneider *et al.*, 2005). In downstream associative auditory fields, studies from both monkeys and humans reported that the border between the anterior and posterior auditory fields ( Figure 1-area PC in the monkey and mSTG in the human) processes pitch attributes that are necessary for the recognition of auditory objects ( Bendor & Wang, 2006). The anterior auditory fields of monkeys were also demonstrated with selectivity for con-specific vocalizations with intra-cortical recordings ( Perrodin *et al.*, 2011; Rauschecker *et al.*, 1995; Russ *et al.*, 2008) and functional imaging ( Joly *et al.*, 2012; Petkov *et al.*, 2008; Poremba *et al.*, 2004). One fMRI monkey study further demonstrated a role of the aSTG in the recognition of individual voices ( Petkov *et al.*, 2008). The role of the human mSTG-aSTG in sound recognition was demonstrated via functional imaging studies that correlated activity in this region with isolation of auditory objects from background noise ( Scheich *et al.*, 1998; Zatorre *et al.*, 2004) and with the recognition of spoken words ( Binder *et al.*, 2004; Davis & Johnsrude, 2003; Liebenthal *et al.*, 2005; Narain *et al.*, 2003; Obleser *et al.*, 2006; Obleser *et al.*, 2007; Scott *et al.*, 2000), voices ( Belin & Zatorre, 2003), melodies ( Benson *et al.*, 2001; Leaver & Rauschecker, 2010), environmental sounds ( Lewis *et al.*, 2006; Maeder *et al.*, 2001; Viceic *et al.*, 2006), and non-speech communicative sounds ( Shultz *et al.*, 2012). A Meta-analysis of fMRI studies ( DeWitt & Rauschecker, 2012) further demonstrated functional dissociation between the left mSTG and aSTG, with the former processing short speech units (phonemes) and the latter processing longer units (e.g., words, environmental sounds). A study that recorded neural activity directly from the left pSTG and aSTG reported that the aSTG, but not pSTG, was more active when the patient listened to speech in her native language than unfamiliar foreign language ( Lachaux *et al.*, 2007-patient 1). Consistently, electro stimulation to the aSTG of this patient resulted in impaired speech perception ( Lachaux *et al.*, 2007-patient 1; see also Matsumoto *et al.*, 2011; Roux *et al.*, 2015 for similar results). Intra-cortical recordings from the right and left aSTG further demonstrated that speech is processed laterally to music ( Lachaux *et al.*, 2007-patient 2). An fMRI study of a patient with impaired sound recognition (auditory agnosia) due to brainstem damage was also shown with reduced activation in areas hR and aSTG of both hemispheres when hearing spoken words and environmental sounds ( Poliva *et al.,* 2015). Recordings from the anterior auditory cortex of monkeys while maintaining learned sounds in working memory ( Tsunada *et al.*, 2011), and the debilitating effect of induced lesions to this region on working memory recall ( Fritz *et al.*, 2005; Stepien *et al.*, 1960; Strominger *et al.*, 1980), further implicate the AVS in maintaining the perceived auditory objects in working memory. In humans, area mSTG-aSTG was also reported active during rehearsal of heard syllables with MEG ( Kaiser *et al.*, 2003) and fMRI ( Buchsbaum *et al.*, 2005). The latter study further demonstrated that working memory in the AVS is for the acoustic properties of spoken words and that it is independent to working memory in the ADS, which mediates inner speech. Working memory studies in monkeys also suggest that in monkeys, in contrast to humans, the AVS is the dominant working memory store ( Scott *et al.*, 2012).

In humans, downstream to the aSTG, the MTG and TP are thought to constitute the semantic lexicon, which is a long-term memory repository of audio-visual representations that are interconnected on the basis of semantic relationships. (See also the reviews by Hickok & Poeppel, 2007 and Gow, 2012, discussing this topic). The primary evidence for this role of the MTG-TP is that patients with damage to this region (e.g., patients with semantic dementia or herpes simplex virus encephalitis) are reported with an impaired ability to describe visual and auditory objects and a tendency to commit semantic errors when naming objects (i.e., semantic paraphasia; Noppeney *et al.*, 2007; Patterson *et al.*, 2007). Semantic paraphasias were also expressed by aphasic patients with left MTG-TP damage ( Dronkers *et al.*, 2004; Schwartz *et al.*, 2009) and were shown to occur in non-aphasic patients after electro-stimulation to this region ( Hamberger *et al.*, 2007; Roux *et al.*, 2015) or the underlying white matter pathway ( Duffau, 2008). Two meta-analyses of the fMRI literature also reported that the anterior MTG and TP were consistently active during semantic analysis of speech and text ( Binder *et al.*, 2009; Vigneau *et al.*, 2006); and an intra-cortical recording study correlated neural discharge in the MTG with the comprehension of intelligible sentences ( Creutzfeldt *et al.*, 1989).

In contradiction to the Wernicke-Lichtheim-Geschwind model that implicates sound recognition to occur solely in the left hemisphere, studies that examined the properties of the right or left hemisphere in isolation via unilateral hemispheric anesthesia (i.e., the WADA procedure; Hickok *et al.*, 2008) or intra-cortical recordings from each hemisphere ( Creutzfeldt *et al.*, 1989) provided evidence that sound recognition is processed bilaterally. Moreover, a study that instructed patients with disconnected hemispheres (i.e., split-brain patients) to match spoken words to written words presented to the right or left hemifields, reported vocabulary in the right hemisphere that almost matches in size with the left hemisphere ( Zaidel, 1976). (The right hemisphere vocabulary was equivalent to the vocabulary of a healthy 11-years old child). This bilateral recognition of sounds is also consistent with the finding that unilateral lesion to the auditory cortex rarely results in deficit to auditory comprehension (i.e., auditory agnosia), whereas a second lesion to the remaining hemisphere (which could occur years later) does ( Poeppel, 2001; Ulrich, 1978). Finally, as mentioned earlier, an fMRI scan of an auditory agnosia patient demonstrated bilateral reduced activation in the anterior auditory cortices ( Poliva *et al.,* 2015), and bilateral electro-stimulation to these regions in both hemispheres resulted with impaired speech recognition ( Lachaux *et al.*, 2007-patient 2).
