# Supplementary material for: From where to what: a neuroanatomically based evolutionary model of the emergence of speech in humans
Source: F1000Res. 2017 Sep 20;4:67. Originally published 2015 Mar 13. [Version 3] doi: 10.12688/f1000research.6175.3 (PMC5600004; doi:10.12688/f1000research.6175.3)
Supplement: Peer review history

# Peer review: Referee response for version 2

**DOI:** 10.5256/f1000research.8315.r12035

**Published:** 2017-09-13

**Authors:** Josef Rauschecker

**Affiliations:** 1 Laboratory of Integrative Neuroscience and Cognition, Georgetown University, Washington DC, USA

The author has responded well to my criticisms, and the paper has become more readable. Even though one can still debate some of his suggestions (or speculations), I find the paper worthy of publication now, as it will help to enliven an already lively debate on the evolution of speech and language.

 I only have a few more issues with the References:

 In all fairness, the paper by Rauschecker & Tian (2000) should be mentioned when introducing the concepts of auditory ventral and dorsal streams (AVS, ADS) on p. 4. The idea of parallel ventral and dorsal processing streams in the auditory system was first proposed and developed there and even earlier. I leave it up to the author, which one(s) of the papers below he wants to cite.

 I also wonder if references suggested by the reviewers shouldn’t generally be added to the overall reference list of the revised version. For instance, the papers by Bornkessel-Schlesewsky *et al*., which seem highly relevant here, are still not cited, even though they are mentioned by two reviewers.

I have read this submission. I believe that I have an appropriate level of expertise to confirm that it is of an acceptable scientific standard.

## References

[1] RauscheckerJP: Processing of complex sounds in the auditory cortex of cat, monkey, and man. *Acta Otolaryngol Suppl*.1997;532:34-8 944284210.3109/00016489709126142

[2] RauscheckerJP: Parallel processing in the auditory cortex of primates. *Audiol Neurootol*.3(2-3) :86-103 957537910.1159/000013784

[3] RauscheckerJP: Cortical processing of complex sounds. *Curr Opin Neurobiol*.1998;8(4) :516-21 975165210.1016/s0959-4388(98)80040-8

[4] RauscheckerJPTianB: Mechanisms and streams for processing of "what" and "where" in auditory cortex. *Proc Natl Acad Sci U S A*.2000;97(22) : 10.1073/pnas.97.22.11800 11800-6 10.1073/pnas.97.22.11800 11050212PMC34352

# Peer review: Referee response for version 1

**DOI:** 10.5256/f1000research.6619.r11960

**Published:** 2016-01-18

**Authors:** Michael A Arbib

**Affiliations:** 1 Computer Science Department, University of Southern California, Los Angeles, CA, USA

I see this paper as a first draft of what could become an important contribution to neurally based approaches to the study of the evolution of the human brain’s capacity for language. Its importance is three-fold:

1. It treats the ventral and dorsal streams for both the auditory and visual modalities.
2. It regards monkey calls not in terms of perception alone or production alone but rather in terms of their role in the interaction between two individuals in the context of their environment.
3. It places the ability to ask and answer questions at the heart of language use.

Below, I will offer several comments on how some shortcomings of the current version might be removed in future work by Poliva, but first a disclosure: I have emphasized the role of the two visual streams in relation to both the production and comprehension of language with an emphasis on the role of manual gesture and protosign in language evolution, and in terms of visual perception of what an utterance may be about (Arbib, 2013). By contrast, Bornkessel-Schlesewsky and Schlesewsky (2013) offer hypotheses on the roles of the auditory streams in the perception of sentences of a spoken language, linking them to neurolinguistic data from their lab and others. I have attempted a preliminary synthesis of these approaches (Arbib, 2015). More recently, they have co-authored a review of relevant data on the auditory streams in both monkey and human with the claim that no major evolutionary innovations were required in these streams to make language possible (Bornkessel-Schlesewsky, Schlesewsky, Small, & Rauschecker, 2015) – a claim with which I (and, I suspect, Poliva) would disagree. I hope to support the counter-claim in a forthcoming article in the *Journal of Neurolinguistics. *I believe Poliva’s assessment of these articles would enrich his work, but now let me turn to other issues.

1. I endorse the key points of Amy Poremba’s review: (i) The dorsal auditory stream was over-emphasized at the expense of assessing the role of the ventral stream and how these streams are integrated. (ii) Poremba notes the relevance of work from Mishkin’s lab on auditory memory – see, e.g., Fritz, Mishkin, and Saunders (2005) which “raises the possibility that language is unique to humans not only because it depends on speech but also because it requires long-term auditory memory.” I would add that Aboitiz and his colleagues have emphasized the expansion of working memory capacity as a key element in evolving a language-ready brain (see Aboitiz, 2012, for a recent review of this approach). (iii) The leap from contact calls to “individuals … capable of inventing new words and offspring … capable of inquiring about objects in their environment and learning their names via mimicry” is essentially unbridged.
2. Since there are many monkey calls, it seems unclear why, if one is to use these calls as the core for evolving a brain with language, one should focus on contact calls alone. Including other calls might add more “evolutionary opportunities.” In this regard, note the argument of Seyfarth and Cheney that one may see the structure of language prefigured in the “rules” monkeys develop for social cognition (Cheney & Seyfarth, 2005; Seyfarth & Cheney, 2014).
3. I suspect that further work in language evolution will reveal a “mosaic” of innovations, some of which are apparent in different monkey or ape species. One may hope that studies of the brains of different species will reveal diverse cues that illuminate, perhaps, the convergent evolution of different tiles of the language-supporting neural mosaic of the human brain. Consider, for example, the capability for turn taking in geladas (Gustison, le Roux, & Bergman, 2012; Richman, 1987) and marmosets (Miller, Thomas, Nummela, & de la Mothe, 2015; Takahashi, Narayanan, & Ghazanfar, 2013) as just one of the diverse components of language-ready brain that are differentially evident in different species of nonhuman primate.
4. Figure 1 shows dual stream connectivity between the auditory cortex and frontal lobe of monkeys and humans. What can be said about the intersection of the 2 streams in VLPFC? And what can be said about the interaction of DLPFC and VLPFC? Figure 2 depicts the “From Where to What model” via three stages of neuroanatomical modifications. It might be useful to first provide a diagram focusing on VVS and VDS (initial V for Visual) and discussing the relation in both anatomy and function of these paths with each other. It might also be helpful to present pieces of the model along with the exposition of the related data, postponing this integrative figure until the pieces are in place.
5. A valuable feature of Poliva’s model is its suggestion of how the response to an auditory call might initiate visual search as the basis for action (he emphasizes the mother emitting a call if the child is not seen; a related scenario would be movement toward the child if it were seen). This issue of integration of communication and action, which may (but need not) integrate audition with vision, is an important feature which too few studies take into account. My question is whether he unduly emphasizes cortical pathways involving the frontal eye fields and shortchanges subcortical interactions involving the superior colliculus (noting of course that these are open to cortical influences modulated by the basal ganglia).
6. In Figure 2, Poliva asserts: (i) “Approximately 2.5 million years ago, the *Homo* genus emerged as *a result of* [my italics] duplication of the IPS and subsequent duplication of its frontal projections” (a) Surely, many more changes led to the emergence of *Homo. *(b) At the end of Section 7, Poliva suggests the relevance of endocast data to this claim. Are there relevant data on apes that could help us assess this transition? (ii) “Since the auditory cortex targeted the more proximal of these duplicated parietal regions, a new pathway dedicated for auditory processing emerged (i.e., auditory dorsal stream; ADS.” But monkey data show an ADS, so what is the transition being suggested here? Picking up on the issue in (5), one needs to better understand the division of labor between ADS and subcortical mechanisms (as well as AVS, to reiterate Poremba’s point).
7. Poliva claims to review “evidence for a role of the ADS in the transition from mediating contact calls into mediating human speech” but simply cites data correlating ADS impairment with disorders like speech apraxia. Nothing in the data privileges contact calls over other vocal productions – and, anyway, clear articulation is a far cry [sic] from mechanisms supporting the role of syntax and semantics in language production and perception.
8. In relation to 6(i), Poliva notes the dual role of the parietal lobe in sensory-motor transformation of both audio-spatial and verbal information, and proposes that during *Hominin* evolution there was a cortical field duplication, of the IPS with further duplication of its projections to the VLPFC which resulted in a pathway dedicated for audio-vocal conversion. How would this serve people who employ a signed language? (Of course, those who advocate a gestural origin of language must face the complementary question of how visuo-manual pathways came to support audio-vocal signals – which they must do because other primates lack vocal learning, let alone the use of syntax and semantics in either domain.)
9. Poliva stresses that the ability to ask and answer questions is an essential feature of language use. I agree. Future work on language evolution should pay more attention to the challenge of explaining how this evolved. However the focus on modifying contact calls with prosodic intonations seems to me too narrow (I may be wrong, but more argument would be needed) and (as Poremba observed) the account of the transition remains too sketchy. Poliva cites “the ability of present-day infants of using intonations for changing the pragmatic utilization of a word from a statement to a command/demand (“mommy!”) or a question (“mommy?”),” but one must be careful to distinguish these infant “communicative acts” from the ability to deploy grammar to formulate an open-ended repertoire of commands and questions using the structures of a language – let along being able to marshal answers to questions of even modest complexity.
10. In any case, it seems mistaken to place exclusive emphasis on the role of ADS in the transition – one might thus assess the hypotheses of Bornkessel-Schlesewsky and Schlesewsky (2013) on the roles of both ADS and AVS (and frontal areas) in speech comprehension. However, a companion paper is promised: “Discussing the transition from exchanging low-level distress contact calls into complex vocal language, however, is beyond the scope of the present paper and a model for such transition is discussed [at] length in a sibling paper titled ‘Vocal Mimicry as the Sculptor of the Human Mind. A Neuroanatomically based Evolutionary Model of The Emergence of Vocal Language’ (Poliva, in preparation).” Perhaps it would be better if less were said about this topic in the present paper so that the implications of the evidence on ADS function and evolution could be better assessed for their merits irrespective of the contact call hypothesis.

I have read this submission. I believe that I have an appropriate level of expertise to confirm that it is of an acceptable scientific standard, however I have significant reservations, as outlined above.

## References

[1] AboitizF: Gestures, vocalizations, and memory in language origins. *Front Evol Neurosci*.2012;4: 10.3389/fnevo.2012.00002 2 10.3389/fnevo.2012.00002 22347184PMC3269654

[2] ArbibMA: Mirror Systems and the Neurocognitive Substrates of Bodily Communication and Language. In C. Müller, A. Cienki, E. Fricke, S. Ladewig, D. McNeill. *Body-Language Communication*.2013;445-460

[3] ArbibMA: Towards a Computational Comparative Neuroprimatology: Framing the language-ready brain. *Phys Life Rev*.2015; 10.1016/j.plrev.2015.09.003 10.1016/j.plrev.2015.09.003 26482863

[4] Bornkessel-SchlesewskyISchlesewskyM: Reconciling time, space and function: a new dorsal-ventral stream model of sentence comprehension. *Brain Lang*.2013;125(1) : 10.1016/j.bandl.2013.01.010 60-76 10.1016/j.bandl.2013.01.010 23454075

[5] Bornkessel-SchlesewskyISchlesewskyMSmallSLRauscheckerJP: Neurobiological roots of language in primate audition: common computational properties. *Trends Cogn Sci*.2015;19(3) : 10.1016/j.tics.2014.12.008 142-50 10.1016/j.tics.2014.12.008 25600585PMC4348204

[6] CheneyDSeyfarthR: Constraints and preadaptations in the earliest stages of language evolution. *The Linguistic Review*.2005;22(2-4) : 10.1515/tlir.2005.22.2-4.135 10.1515/tlir.2005.22.2-4.135

[7] FritzJMishkinMSaundersRC: In search of an auditory engram. *Proc Natl Acad Sci U S A*.2005;102(26) : 10.1073/pnas.0503998102 9359-64 10.1073/pnas.0503998102 15967995PMC1166637

[8] GustisonMLle RouxABergmanTJ: Derived vocalizations of geladas (Theropithecus gelada) and the evolution of vocal complexity in primates. *Philos Trans R Soc Lond B Biol Sci*.2012;367(1597) : 10.1098/rstb.2011.0218 1847-59 10.1098/rstb.2011.0218 22641823PMC3367700

[9] MillerCTThomasAWNummelaSUde la MotheLA: Responses of primate frontal cortex neurons during natural vocal communication. *J Neurophysiol*.2015;114(2) : 10.1152/jn.01003.2014 1158-71 10.1152/jn.01003.2014 26084912PMC4725106

[10] RichmanB: Rhythm and melody in gelada vocal exchanges. *Primates*.1987;28(2) : 10.1007/BF02382570 199-223 10.1007/BF02382570

[11] SeyfarthRMCheneyDL: The evolution of language from social cognition. *Curr Opin Neurobiol*.2014;28: 10.1016/j.conb.2014.04.003 5-9 10.1016/j.conb.2014.04.003 24813180

[12] TakahashiDYNarayananDZGhazanfarAA: Coupled oscillator dynamics of vocal turn-taking in monkeys. *Curr Biol*.2013;23(21) : 10.1016/j.cub.2013.09.005 2162-8 10.1016/j.cub.2013.09.005 24139740

# Peer review: Referee response for version 1

**DOI:** 10.5256/f1000research.6619.r7964

**Published:** 2015-12-24

**Authors:** Josef Rauschecker

**Affiliations:** 1 Laboratory of Integrative Neuroscience and Cognition, Georgetown University, Washington DC, USA

This is an interesting contribution to the literature on language evolution. The first two sections ('Introduction' and 'Models of Language Processing in the Brain...') are a joy to read. Later sections are more controversial and contain serious flaws that have to be brought up to speed with the current literature. These concerns are summarized here:

1) The terminology is quite fuzzy. For instance, when the author refers to 'perception' he seems to mean 'detection' or 'processing'. In most people's minds, and in most extant models of perception and action, perception is specifically tied to the ventral stream. Therefore, it can, almost by definition, not also be a property of the dorsal stream. This is best exemplified in the Abstract: The author states: *'I propose that the primary role of the auditory dorsal stream (ADS) in monkeys/apes is the perception and response to contact calls.'* This misstatement can be fixed by replacing 'perception' with 'detection'. Similarly, in a later sentence ('Perception of contact calls occurs by the ADS detecting a voice...'), 'Perception' can be substituted by 'Processing'. Thirdly, in the Abstract's second paragraph, the following sentence does not make any sense: 'Because the human ADS processes also speech production and repetition...'. Here, 'processes' needs to be replaced with 'performs'.

2) In the third section, the author first makes a strong case for a role of the ADS in auditory spatial processing, for encoding of sound location in memory and for use of this information in guiding eye movements. The published literature is well represented, though a key reference is missing here ( Tian et al., Science, 2001). Then, in a surprising turnaround, the author suddenly concludes that 'audiospatial input is first converted into a visuospatial code and then processed via a visuospatial network'. The evidence cited stems from 15-year old studies of monkey area LIP, which is part of a visuospatial network; auditory signals, however, are relayed to a different part of IPS (area VIP; Lewis & VanEssen, 2000), for which corresponding studies have not been performed. Figure 2, which pertains to this section, reflects this misinterpretation: While the version on the left is neuroanatomically acceptable (with the only difference that parietal cortex is not just a visuospatial but a multisensory or amodal network, the versions in the center and on the right are incorrect on multiple grounds, most notably by postulating the 'duplication of the IPS [pivoting around an imaginary blue asterisk] and subsequent duplication of its frontal projections'. The assumptions about anatomical connections of the IPL with ventrolateral prefrontal cortex (VLPFC) are largely unsubstantiated and the characterization of VLPFC as a motor representation is plain wrong. I assume what the author may be referring to is ventral premotor cortex (PMv), which is indeed the terminal point of the auditory dorsal stream and is closely interfacing with Broca’s area.

3)  According to a third hypothesis put forward by the author, " *the Homo genus emerged as a result of duplicating the IPS and its frontal projections. This duplication resulted with area Spt and its projections to the VLPFC. In contrast to the visual dorsal stream that processes audiovisual spatial properties, the human ADS processes inner and outer speech*." This hypothesis is seriously flawed, because both ADS and VDS process spatial properties and both process sensorimotor signals. In fact, they may be one and the same structure. Thus, there is no fundamental difference between visual and auditory processing that would require duplication of IPS or its projections or special evolution of speech (see Bornkessel et al., 2015).

4) Additional citations that the author should add:

DeWitt and Rauschecker, 2012 ^1^

DeWitt and Rauschecker, 2013 ^2^

Bornkessel-Schlesewsky, *et al*., 2015 ^3^

Mesulam, *et al*., 2015 ^4^

Roux, *et al*., 2015 ^5^

I have read this submission. I believe that I have an appropriate level of expertise to confirm that it is of an acceptable scientific standard, however I have significant reservations, as outlined above.

## References

[1] DeWittIRauscheckerJP: Phoneme and word recognition in the auditory ventral stream. *Proc Natl Acad Sci U S A*.2012;109(8) : 10.1073/pnas.1113427109 E505-14 10.1073/pnas.1113427109 22308358PMC3286918

[2] DeWittIRauscheckerJ: Wernicke’s area revisited: Parallel streams and word processing. *Brain and Language*.2013;127(2) : 10.1016/j.bandl.2013.09.014 181-191 10.1016/j.bandl.2013.09.014 PMC409885124404576

[3] Bornkessel-SchlesewskyISchlesewskyMSmallSLRauscheckerJP: Neurobiological roots of language in primate audition: common computational properties. *Trends Cogn Sci*.2015;19(3) : 10.1016/j.tics.2014.12.008 142-50 10.1016/j.tics.2014.12.008 25600585PMC4348204

[4] MesulamMMThompsonCKWeintraubSRogalskiEJ: The Wernicke conundrum and the anatomy of language comprehension in primary progressive aphasia. *Brain*.2015;138(Pt 8) : 10.1093/brain/awv154 2423-37 10.1093/brain/awv154 26112340PMC4805066

[5] RouxFEMiskinKDurandJBSackoORéhaultETanovaRDémonetJF: Electrostimulation mapping of comprehension of auditory and visual words. *Cortex*.2015;71: 10.1016/j.cortex.2015.07.001 398-408 10.1016/j.cortex.2015.07.001 26332785

# Response

**Published:** 2016-01-05

**Authors:** Oren Poliva

**Affiliations:** Bangor University, UK

This is an interesting contribution to the literature on language evolution. The first two sections ('Introduction' and 'Models of Language Processing in the Brain...') are a joy to read. Later sections are more controversial and contain serious flaws that have to be brought up to speed with the current literature. These concerns are summarized here:

1) The terminology is quite fuzzy. For instance, when the author refers to 'perception' he seems to mean 'detection' or 'processing'. In most people's minds, and in most extant models of perception and action, perception is specifically tied to the ventral stream. Therefore, it can, almost by definition, not also be a property of the dorsal stream. This is best exemplified in the Abstract: The author states: *'I propose that the primary role of the auditory dorsal stream (ADS) in monkeys/apes is the perception and response to contact calls.'* This misstatement can be fixed by replacing 'perception' with 'detection'. Similarly, in a later sentence ('Perception of contact calls occurs by the ADS detecting a voice...'), 'Perception' can be substituted by 'Processing'. Thirdly, in the Abstract's second paragraph, the following sentence does not make any sense: 'Because the human ADS processes also speech production and repetition...'. Here, 'processes' needs to be replaced with 'performs'.

**Response:** **As far as I understand it, perception refers to all elements of the external world that reach our awareness. In accordance with this definition, through the AVS we perceive the identity of sounds and through the ADS we perceive the location of sounds. As human speech production is also processed in the ADS, I would expect that we also perceive elements of speech preparation through the ADS. A good example is a study that reported of patients who were electrically stimulated in the left inferior parietal lobule and consequently believed they produced sounds, when in fact they didn’t (Desmurget et al., 2009). This study can be argued to demonstrates perception of speech preparation in the ADS. Nonetheless, considering that different researchers might have different definitions for perception, I replaced instances that describe perception with detection wherever it was applicable.**

**Desmurget M, Reilly KT, Richard N, Szathmari A, Mottolese C, Sirigu A. Movement intention after parietal cortex stimulation in humans. Science. 2009 May 8;324(5928):811–3.**

2) In the third section, the author first makes a strong case for a role of the ADS in auditory spatial processing, for encoding of sound location in memory and for use of this information in guiding eye movements. The published literature is well represented, though a key reference is missing here ( Tian et al., Science, 2001). Then, in a surprising turnaround, the author suddenly concludes that 'audiospatial input is first converted into a visuospatial code and then processed via a visuospatial network'. The evidence cited stems from 15-year old studies of monkey area LIP, which is part of a visuospatial network; auditory signals, however, are relayed to a different part of IPS (area VIP; Lewis & VanEssen, 2000), for which corresponding studies have not been performed. Figure 2, which pertains to this section, reflects this misinterpretation: While the version on the left is neuroanatomically acceptable (with the only difference that parietal cortex is not just a visuospatial but a multisensory or amodal network, the versions in the center and on the right are incorrect on multiple grounds, most notably by postulating the 'duplication of the IPS [pivoting around an imaginary blue asterisk] and subsequent duplication of its frontal projections'.  …. According to a third hypothesis put forward by the author, " *the Homo genus emerged as a result of duplicating the IPS and its frontal projections. This duplication resulted with area Spt and its projections to the VLPFC. In contrast to the visual dorsal stream that processes audiovisual spatial properties, the human ADS processes inner and outer speech*." This hypothesis is seriously flawed, because both ADS and VDS process spatial properties and both process sensorimotor signals. In fact, they may be one and the same structure. Thus, there is no fundamental difference between visual and auditory processing that would require duplication of IPS or its projections or special evolution of speech (see Bornkessel et al., 2015).

**Response:** **Although I don’t entirely agree with the reviewer’s perspective in this regard, given that the paper is already rich in evidence and hypotheses, I removed the sections (last paragraph of section 3 and section 7) discussing these hypotheses in the revised version. Also, I removed figure 2 from the revised version, and accordingly modified the manuscript to accommodate this change.**

3) …I assume what the author may be referring to is ventral premotor cortex (PMv), which is indeed the terminal point of the auditory dorsal stream and is closely interfacing with Broca’s area.

**Response:** **Thinking back, I agree with the reviewer that referring to this region as the ‘ventral premotor cortex’ is more accurate. The reason I referred to this region as the ventrolateral prefrontal cortex is to be consistent with previous papers (e.g., Romansky et al., 1999). As it possible that the area most often referred to as Broca’s area encompasses both parts of the ventrolateral prefrontal cortex and ventral premotor cortex, in the revised manuscript I replaced the term ‘ventrolateral prefrontal cortex’ with its anatomical equivalent, the ‘inferior frontal gyrus’.**

**Romanski LM, Bates JF, Goldman-Rakic PS. Auditory belt and parabelt projections to the prefrontal cortex in the rhesus monkey. J Comp Neurol. 1999 Jan 11;403(2):141–57.**

**As a final note, I want to thank the reviewer for his time and effort, and hope he finds the revised version even more enjoyable to read.**

# Peer review: Referee response for version 1

**DOI:** 10.5256/f1000research.6619.r8933

**Published:** 2015-07-17

**Authors:** Amy Poremba

**Affiliations:** 1 Department of Psychology, University of Iowa, Iowa City, IA, USA

This contribution is a wide-ranging theory of how speech evolved in humans, which incorporates the dorsal and ventral auditory processing streams, but primarily focused on the auditory dorsal stream.  

There are several large leaps in the proposed trajectory for language evolution such as, “eventually, individuals were capable of inventing new words and offspring were capable of inquiring about objects in their environment and learning their names via mimicry.” While the first part of the overall proposed theory is well supported, these latter stages are under-supported by current knowledge, particularly when moving to discussing individuals that became capable of enunciating novel calls (e.g., last paragraph of introduction); (some publications that may be helpful, comment by Meguerditchian *et al.*, 2014; original article, Ackermann *et al.*, 2014). The steps proposed for inventing new words and inquiring about objects are likely to require a large number of processes and the theory does not specify what those steps might be. Overall, Poliva’s theory as set forth does generate some interesting, testable, hypotheses as demonstrated in section 9, and the leaps in the logical flow do not negate these as the hypotheses are more closely related to the current knowledge base.

As this is a theory of “From where to what,” missing for me was a better description of how the dorsal and ventral streams might interact in this theory. Calls still need to be “recognized” as auditory objects and imaging and recording studies have indicated the ventral stream does process this type of information. The ventral stream was given much less prominence and described in the appendix. It would be nice to include a paragraph or two on how the two systems may work together or how the ventral stream object identification comes to participate or interact with word formation and questions about objects.

In the first paragraph of the introduction, curiosity toward the unknown may be related to non-human primates’ tendency to pick novel objects from known objects. This is also true in many lower animals. The development of curiosity of objects that are absent from our environment as Poliva suggests must also be related to memory development. One must be able to remember that objects exist and have detailed memories in order to determine if an object is indeed missing. There are aspects of work by Mishkin and colleagues suggesting that the lack of robust, or expansive, long-term auditory memory may relate to the absence of complex communication systems in non-human primates, such as rhesus macaques. Clearly, visual memory is much more extensive and robust than auditory memory and the sign language that other non-human primates have demonstrated may be related to the robust nature of visual memory. The issue of memory mechanisms necessary for identifying that auditory objects are indeed missing from the environment, and how these may differ and interact between auditory and visual systems, should at least be mentioned in passing.

I have read this submission. I believe that I have an appropriate level of expertise to confirm that it is of an acceptable scientific standard, however I have significant reservations, as outlined above.

# Response

**Published:** 2016-01-05

**Authors:** Oren Poliva

**Affiliations:** Bangor University, UK

This contribution is a wide-ranging theory of how speech evolved in humans, which incorporates the dorsal and ventral auditory processing streams, but primarily focused on the auditory dorsal stream.  

There are several large leaps in the proposed trajectory for language evolution such as, “eventually, individuals were capable of inventing new words and offspring were capable of inquiring about objects in their environment and learning their names via mimicry.” While the first part of the overall proposed theory is well supported, these latter stages are under-supported by current knowledge, particularly when moving to discussing individuals that became capable of enunciating novel calls (e.g., last paragraph of introduction); (some publications that may be helpful, comment by Meguerditchian *et al.*, 2014; original article, Ackermann *et al.*, 2014). The steps proposed for inventing new words and inquiring about objects are likely to require a large number of processes and the theory does not specify what those steps might be. Overall, Poliva’s theory as set forth does generate some interesting, testable, hypotheses as demonstrated in section 9, and the leaps in the logical flow do not negate these as the hypotheses are more closely related to the current knowledge base.

**Response:** **I agree with the reviewer that the final evolutionary stages show a leap and are not strongly substantiated with evidence. As mentioned in the paper, in depth discussion of these stages is presented in a sibling paper, which is currently in writing. Nonetheless, in the revised manuscript, I made more effort to describe possible transition to mimicry. Moreover, I removed discussing this issue from the abstract and introduction, as it is not the primary concern of the present paper.**

As this is a theory of “From where to what,” missing for me was a better description of how the dorsal and ventral streams might interact in this theory. Calls still need to be “recognized” as auditory objects and imaging and recording studies have indicated the ventral stream does process this type of information. The ventral stream was given much less prominence and described in the appendix. It would be nice to include a paragraph or two on how the two systems may work together or how the ventral stream object identification comes to participate or interact with word formation and questions about objects.

**Response:** **As mentioned above, in the revised manuscript I downplayed the role of the ADS in object naming and mimicry, and limited the discussion to speech. In depth discussion into the role of the AVS in these functions will be presented in the sibling paper. There was simply too many hypotheses and topics to cover, which made it impossible to include them all in a single paper.**

In the first paragraph of the introduction, curiosity toward the unknown may be related to non-human primates’ tendency to pick novel objects from known objects. This is also true in many lower animals.

**Response:** **The hypothesis that curiosity to novel objects prompted our curiosity to the unknown is an interesting alternative hypothesis. Humans, however, since the beginning of written history, were also documented with another curiosity: desire to explore unknown places. In the present paper, I present evidence that the primary drive for the emergence of speech was by lost infants and mothers seeking to reunite. This model seems to explain both the emergence of speech and our unique curiosity for the unknown and is thus parsimonious. Presenting an alternative explanation would entail evidence for a different evolutionary course, and is thus beyond the scope of the present paper. Saying that, I’ll be very interested to read about evidence for an evolutionary course that explains the curiosity to the unknown from this perspective. In the present model, I argue that the first question ever asked was “where are you”. It leaves me wondering that if the curiosity to the unknown was prompted by curiosity to novel objects, then what could have been the first question?**

The development of curiosity of objects that are absent from our environment as Poliva suggests must also be related to memory development. One must be able to remember that objects exist and have detailed memories in order to determine if an object is indeed missing. There are aspects of work by Mishkin and colleagues suggesting that the lack of robust, or expansive, long-term auditory memory may relate to the absence of complex communication systems in non-human primates, such as rhesus macaques. Clearly, visual memory is much more extensive and robust than auditory memory and the sign language that other non-human primates have demonstrated may be related to the robust nature of visual memory. The issue of memory mechanisms necessary for identifying that auditory objects are indeed missing from the environment, and how these may differ and interact between auditory and visual systems, should at least be mentioned in passing.

**Response** **: The hypothesis that expansion of auditory memory contributed to the development of language is very interesting and I do appreciate that the reviewer brought this research to my attention. However, in the present paper I only describe an evolutionary course up to the advent of the first conversation. Enhancement of auditory memory likely occurred in later stages of language development, and is thus beyond the scope of the present paper.**

**As a final note, I want to thank the reviewer for her insightful comments and opinions, and hope that she enjoys the revised version of the paper.**
